# Fluid shear stress triggers cholesterol biosynthesis and uptake in inner medullary collecting duct cells, independently of nephrocystin-1 and nephrocystin-4

**DOI:** 10.3389/fmolb.2023.1254691

**Published:** 2023-10-17

**Authors:** Meriem Garfa Traoré, Federica Roccio, Caterina Miceli, Giulia Ferri, Mélanie Parisot, Nicolas Cagnard, Marie Lhomme, Nicolas Dupont, Alexandre Benmerah, Sophie Saunier, Marion Delous

**Affiliations:** ^1^ Laboratory of Hereditary Kidney Disease, INSERM UMR 1163, Imagine Institute, Université Paris Cité, Paris, France; ^2^ Cell Imaging Platform, Structure Fédérative de Recherche Necker, INSERM US24/CNRS UMS3633, Université Paris Cité, Paris, France; ^3^ Institut Necker Enfants-Malades (INEM), INSERM U1151/CNRS UMR 8253, Université Paris Cité, Paris, France; ^4^ Genomics Core Facility, Institut Imagine-Structure Fédérative de Recherche Necker, INSERM U1163 et INSERM US24/CNRS UMS3633, Université Paris Cité, Paris, France; ^5^ Bioinformatic Platform, Institut Imagine-Structure Fédérative de Recherche Necker, INSERM U1163 et INSERM US24/CNRS UMS3633, Université Paris Cité, Paris, France; ^6^ ICAN Omics, IHU ICAN Foundation for Innovation in Cardiometabolism and Nutrition, Pitié-Salpêtrière Hospital, Paris, France

**Keywords:** nephronophthisis, NPHP1, NPHP4, shear stress, cholesterol

## Abstract

Renal epithelial cells are subjected to fluid shear stress of urine flow. Several cellular structures act as mechanosensors–the primary cilium, microvilli and cell adhesion complexes–that directly relay signals to the cytoskeleton to regulate various processes including cell differentiation and renal cell functions. Nephronophthisis (NPH) is an autosomal recessive tubulointerstitial nephropathy leading to end-stage kidney failure before adulthood. *NPHP1* and *NPHP4* are the major genes which code for proteins that form a complex at the transition zone of the primary cilium, a crucial region required for the maintenance of the ciliary composition integrity. These two proteins also interact with signaling components and proteins associated with the actin cytoskeleton at cell junctions. Due to their specific subcellular localization, we wondered whether NPHP1 and NPHP4 could ensure mechanosensory functions. Using a microfluidic set up, we showed that murine inner medullary collecting ductal cells invalidated for *Nphp1* or *Nphp4* are more responsive to immediate shear exposure with a fast calcium influx, and upon a prolonged shear condition, an inability to properly regulate cilium length and actin cytoskeleton remodeling. Following a transcriptomic study highlighting shear stress-induced gene expression changes, we showed that prolonged shear triggers both cholesterol biosynthesis pathway and uptake, processes that do not seem to involve neither NPHP1 nor NPHP4. To conclude, our study allowed us to determine a moderate role of NPHP1 and NPHP4 in flow sensation, and to highlight a new signaling pathway induced by shear stress, the cholesterol biosynthesis and uptake pathways, which would allow cells to cope with mechanical stress by strengthening their plasma membrane through the supply of cholesterol.

## Introduction

Nephronophthisis (NPH) is an autosomal recessive tubulo-interstitial nephropathy that accounts for up to 15% of end stage kidney disease (ESKD) in children (Hamiwka et al., 2008; Salomon et al., 2009). The juvenile form is the most prevalent (48%) and progresses to ESKD before the age of 15 (Hildebrandt et al., 2009; Petzold et al., 2023). Typical clinical symptoms are polyuria, polydipsia with regular fluid intake, impaired sodium reabsorption leading to hypovolemia and hyponatremia, anemia and growth retardation (König et al., 2017; Stokman et al., 2018). Histologically, it is characterized by a thickening of the tubular basement membranes and a massive, diffuse interstitial fibrosis, accompanied by the appearance of tubular cysts at the cortico-medullary junction (Hildebrandt et al., 2009). In about 50% of cases, patients with NPH have extrarenal manifestations defining specific syndromic forms (Petzold et al., 2023). To date, mutations in more than 20 different genes have been identified to cause NPH (Braun and Hildebrandt, 2017; Devlin and Sayer, 2019; Petzold et al., 2023), the major ones being NPHP1 and NPHP4 genes, which account for 53% and 5% of cases, respectively (Petzold et al., 2023).

NPHP genes are ubiquitously expressed, and most of them code for proteins located at the primary cilia, thus classifying NPH as a ciliopathy. Primary cilia are sensory organelles at the cell surface that are essential for the transduction of extracellular chemical and mechanical signals to ensure organogenesis and tissue homeostasis. More specifically, nephrocystin-1 (NPHP1) and nephrocystin-4 (NPHP4) are at the transition zone of the primary cilium, participating in the control of ciliary component entry and exit from the organelle (Jauregui and Barr, 2005; Huang et al., 2011; Garcia et al., 2022). NPHP1 and NPHP4 also localize to cell junctions and interact with components of the adherens junctions (Donaldson et al., 2000; Otto et al., 2000; Benzing et al., 2001; Donaldson et al., 2002; Mollet et al., 2005; Delous et al., 2009); they also interact with tensin and filamin present at focal adhesions, linking them to the actin cytoskeleton (Donaldson et al., 2000; Benzing et al., 2001).

The renal tubular epithelial cells are subjected to the flow of primitive urine, which varies along the tube and applies various forces on the cell surface, such as stretching forces, drag forces and fluid shear stress (Weinbaum et al., 2010; Gilmer et al., 2018). Several cellular structures have been proposed to contribute to flow sensing in the nephron, including microvilli, glycocalyx, focal adhesion, ion channels or transporters, and the primary cilium (Verschuren et al., 2020). All these structures transduce shear stress into biological responses, triggering a cascade of signals (ATP release, intracellular calcium increase, nitric oxide (NO) and reactive oxygen species (ROS) production, prostaglandin synthesis, MAP-kinase dependent signaling activation) (Liu et al., 2003; Simons et al., 2005; Sipos et al., 2009; Cabral, Hong, and Garvin, 2010; Flores et al., 2012; Svenningsen, Burford, and Peti-Peterdi, 2013; Zheleznova et al., 2016b; Sheng et al., 2018) that regulate typical renal functions, such as water and ion reabsorption, cytoskeleton remodeling (Essig et al., 2001; Duan et al., 2008) and cell differentiation (Rodat-Despoix et al., 2013).

Due to their location at strategic mechanosensation points (primary cilium, cell junctions), we investigated the role of NPHP1 and NPHP4 in flow sensing using murine inner medullary collecting duct (mIMCD3) deficient for *Nphp1* (KD_N1) or *Nphp4* (KD_N4). For this, we analyzed the ability of KD_N1 and KD_N4 cells to respond to instantaneous or long exposure (48 h) to shear stress. Moderate alterations could be detected in the regulation of cilium length and actin cytoskeleton remodeling, and by transcriptomic analysis, we revealed that prolonged shear stress activates the cholesterol biosynthesis pathway, with an upregulation of several genes encoding enzymes of the synthesis cascade, confirmed by immunostaining and by lipidomics. We also observed an increased cholesterol uptake. These processes, whether it be cholesterol biosynthesis or uptake, do not seem to involve NPHP1 and NPHP4 proteins.

## Results

### KD_N1 and KD_N4 cell lines are more responsive to immediate shear stress

**FIGURE 1:**
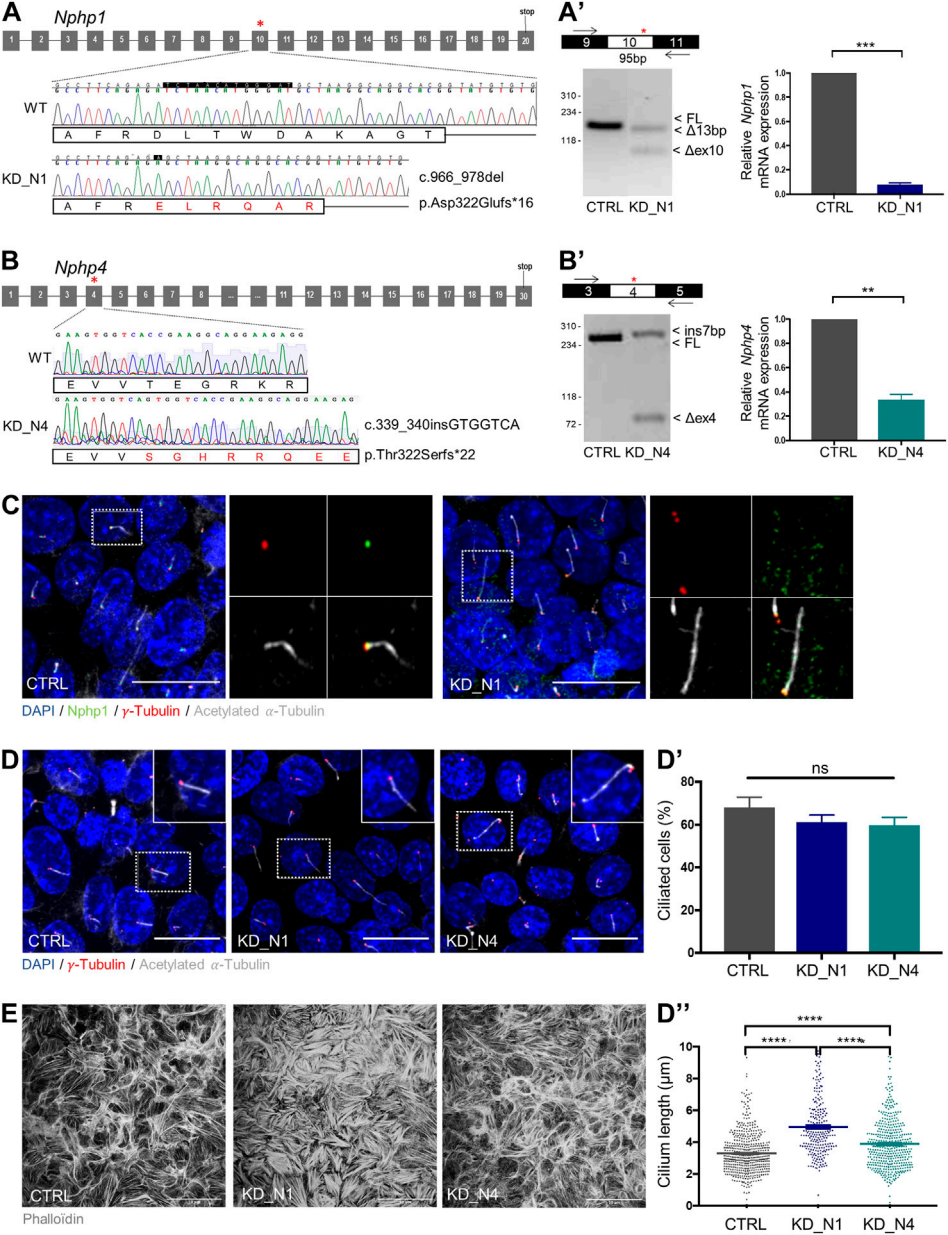
Generation of *Nphp1* (KD_N1) and *Nphp4* (KD_N4)-invalidated mIMCD3 cells by CRISPR-Cas9 technique. **(A, B)** Schemas of *Nphp1* **(A)** and *Nphp4* **(B)** gene structure, with grey boxes representing the exons and black lines the introns. Asterisks indicate the targeted exons (exon 10 in *Nphp1*, exon 4 in *Nphp4*), in which two guides were designed to use the double nickase Cas9 strategy. Chromatograms show the wild-type and mutated sequences of selected clones. For the KD_N1 clone, a homozygous frameshift deletion of 13 base pairs (c.966_978delTCTAACATGGGAT) was selected which leads to a premature stop codon in exon 11 (p.Asp322Glufs*16). For the KD_N4 clone, a homozygous frameshift insertion of 7 base pairs (c.339_340insGTGGTCA) was selected which results into a premature stop codon in exon 4 (p.Thr322Serfs*22). RT-PCR and RT-qPCR **(A′, B′)** analyses of the impact of the indel mutations on the splicing of the CRISPR/Cas9-targeted exons and on the total mRNA expression of Nphp genes relative to that of Hprt in CTRL, KD_N1 **(A′)** and KD_N4 **(B′)** cell lines. Graphs **(A′–B′)** show the mean ± SEM of *n* = 3 independent experiments. ***p = 0,0006, **p = 0,0079, using Mann-Whitney test. **(C**) Immunostaining of CTRL and KD_N1 cells with anti-NPHP1 $\left(\text{green}\right)$ , γ -tubulin (basal bodies, red) and acetylated α -tubulin (cilia, grey) antibodies and DAPI (nuclei, blue). Scale bars, 20 μm. Higher magnification images of the outlined area are shown on the right. **(D)**- Immunostaining of primary cilia in CTRL, KD_N1 and KD_N4 cells, cultured on slides, using γ -tubulin (basal bodies, red) and acetylated α -tubulin (cilia, grey) antibodies and DAPI (nuclei, blue). Graphs show the quantification of the percentage of ciliated cells **(D′)** and cilium length **(D′′)** of n > 350 analyzed cells issued from 3 independent experiments. Bars represent the mean ± SEM. ns: not significant, ****p < 0.0001, using One Way Anova test. Scale bars, 20 µm. **(E)** Phalloidin immunostaining in CTRL, KD_N1 and KD_N4 cells cultured on slides. Scale bars, 50 µm .

To assess the role of NPHP1 and NPHP4 in urine flow sensation, we first generated mIMCD3 cellular models depleted for *Nphp1* and *Nphp4* using the CRISPR-Cas9 technique. For each line, a homozygous frameshift mutation that partially induced an out-of-frame exon skipping was selected (Figures 1A–B’). By RT-qPCR, we observed a partial RNA decay of mutated *Nphp1* (8% left) and *Nphp4* (34% left) transcripts (Figures 1A’, B’), and by immunofluorescence, a partial loss of Nphp1 at the transition zone (Figure 1C). The lack of anti-Nphp4 antibody prevented us to analyse the expression of the protein. Assuming that there could be expression of partially functional proteins left, we hereafter named the cell lines KD_N1 and KD_N4.

To validate the deleterious effect of the selected mutations at cellular level, we first analyzed ciliogenesis, known to be altered in other NPHP model (Delous et al., 2009; Burcklé et al., 2011; Vincensini et al., 2011; Li et al., 2021; Garcia et al., 2022). No change of percentage of ciliated cells was observed; however, cilium length was increased in KD_N1 and KD_N4 cell lines (Figures 1D–D”). We also analyzed the actin cytoskeleton network. In both cell lines, and especially in KD_N1 cells, the network was altered with an accumulation of thick stress fibers (Figure 1E), confirming the potential roles of NPHP1 and NPHP4 in actin cytoskeleton regulation (Donaldson et al., 2000; Benzing et al., 2001).

**FIGURE 2:**
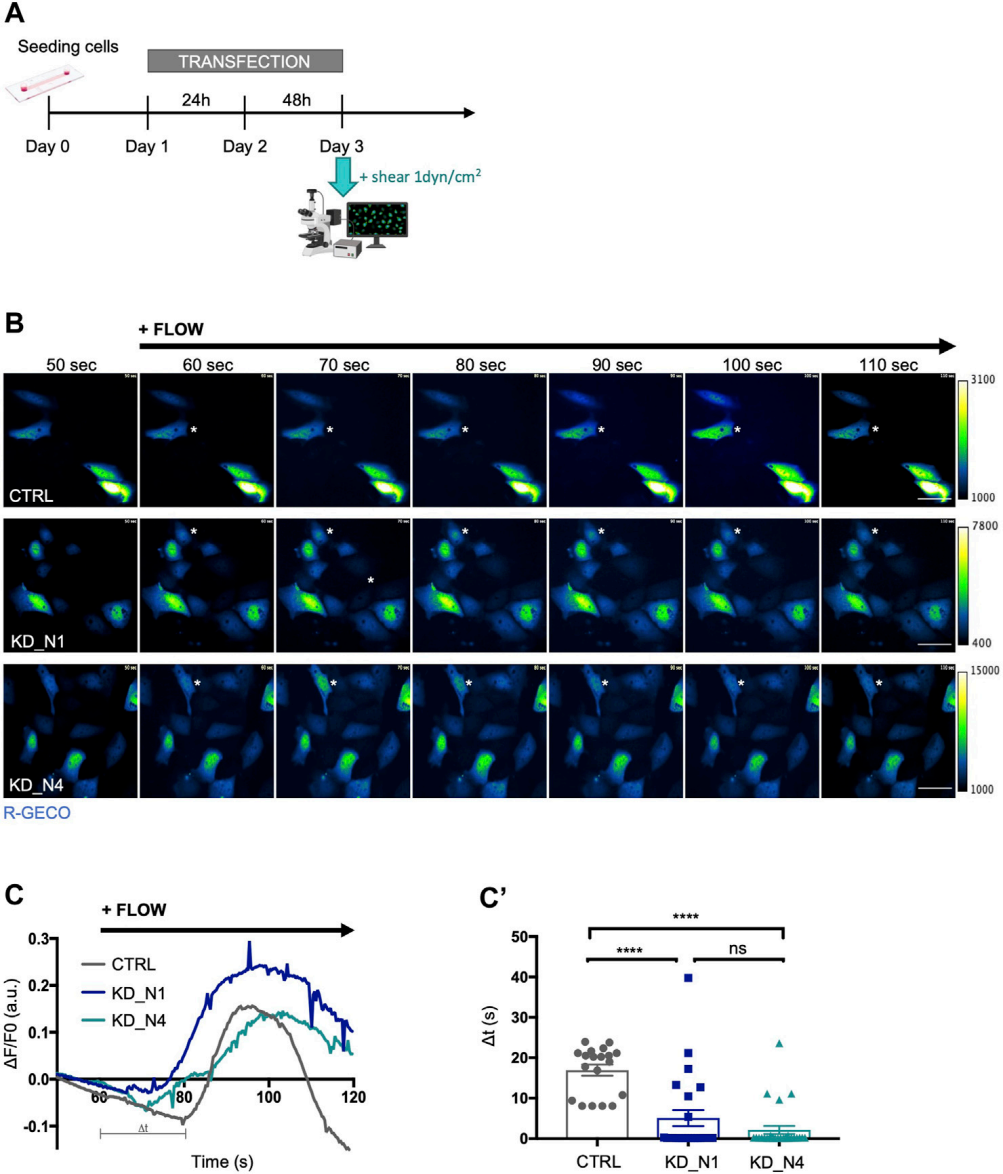
KD_N1 and KD_N4 cell lines are more responsive to immediate shear stress. **(A)** Experimental timeline for live shear stress imaging. 200,000 cells were seeded at day 0. On day 1, the cells were transfected with the plasmid containing the calcium biosensor R-GECO for 48 h. On day 3, the whole system was transferred next to the microscope to allow visualization of the calcium response following the application of a 1dyn/cm^2^ shear. **(B)** Representative pseudo-colored images of the calcium biosensor R-GECO fluorescence, issued from movies of CTRL and KD cells subjected to a physiological shear stress of 1 dyn/cm^2^ during a videomicroscopy of 120 s. The shear stress was applied after 60 s of recording. Of note, since the R-GECO biosensor was transiently transfected, the level of expression of the biosensor, and thus the intensity of its fluorescence, is not interpreted, as it may not be equivalent in each single cell analysed, explaining the different scales of the colour map. Dark blue represents low level of Ca2+, green intermediate level of Ca2+ and white denotes a high level of Ca2+. Asterisks point to representative cells that react to shear. Scale bars: 50 µm. **(C)** R-GECO signal intensity normalized to background $\left(\Delta F/F0\right)$ *,* where F0 is the average intensity over the first 30 s. The graph shows the outline of R-GECO activation of one representative experiment. Δt represents the response time following shear stress initiation. Quantifications of Δt **(C′)** of each single cell analysed are plotted in graphs, which represent the mean of *n* > 30 cells from 3 independent experiments. ns: not significant, ****p < 0.0001, using Kruskal–Wallis test. a.u.: arbitrary unit.

To subject cells to a laminar flow, we used a microfluidic system (Ibidi) and apply a physiological shear stress of 1 dyn/cm^2^. Shear stress is known to induce an immediate calcium response in renal tubular cells. Thus, we transiently transfected cells with the calcium biosensor R-GECO (Zhao et al., 2011) and monitored instantaneous cytosolic calcium response by videomicroscopy for 5 min during the onset of shear stress (Figure 2A), that was applied after 1 min of recording. An increase of the fluorescence intensity of R-GECO was observed in all transfected cells of all cell lines; however, the response occurred around 17 s after flow onset in control cells, whereas it occurred faster, after about 5 s in KD_N1 cells and 2 s in KD_N4 cells (Figures 2B−C’; Supplementary Movies). Of note, as the transfection was transient with variable R-GECO expression, the maximum of fluorescence intensity was not interpreted. Therefore, we concluded that KD_N1 and KD_N4 cells were more responsive to shear stress, with a faster immediate calcium response than control cells.

### KD_N1 and KD_N4 cells exhibit moderate response alterations to prolonged shear stress

In order to analyze whether the sensibility of KD_N1 and KD_N4 cell lines to shear stress persisted after a long exposure to flow, we decided to study several cellular processes after 48 h of shear stress (Supplementary Figure S1).

**FIGURE 3:**
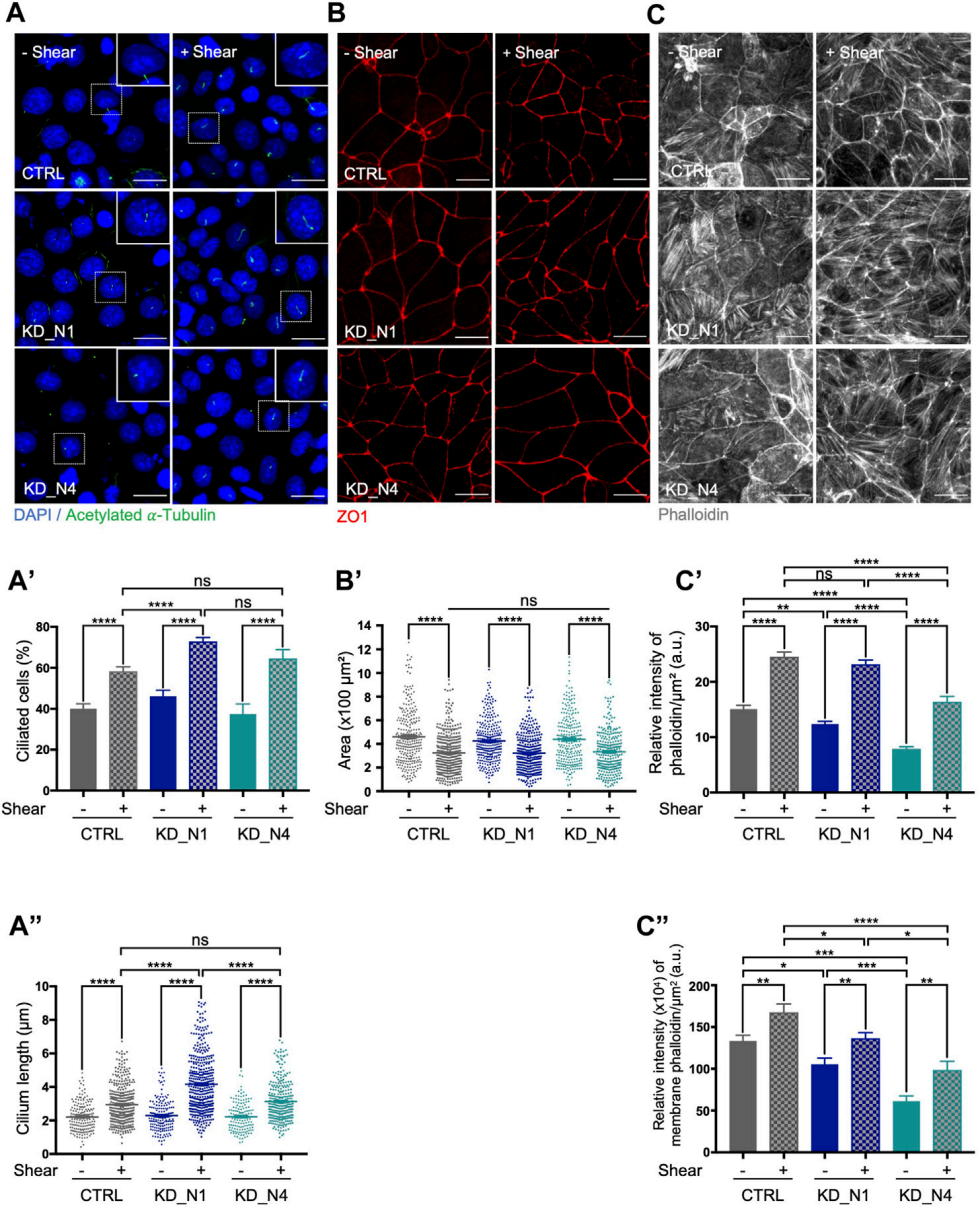
KD_N1 and KD_N4 cells exhibit moderate response alterations to prolonged shear stress of 48 h. **(A)** Ciliogenesis in static condition (–Shear) and after shear stress (+Shear) was analysed by immunofluorescence in CTRL, KD_N1 and KD_N4 cells labelled with anti-acetylated α -tubulin antibody (cilia, green) and DAPI (nuclei, blue). Higher magnification images of the outlined area are shown on the right. Graphs represent the quantification of the percentage of ciliated cells **(A′)** and cilium length **(A”)** of n > 350 cells from 3 independent experiments. Bars indicate the mean ± SEM. ns: not significant, ****p < 0.0001, ***p < 0,0003, using unpaired *t*-test for a 2 by 2 comparison (Shear vs. Static) or One way Anova test (CTRL vs. KD cells). Scale bars: 20 µm. **(B)** Cell area in static condition (–Shear) and after shear stress (+Shear) was analysed by immunofluorescence in CTRL, KD_N1 and KD_N4 cells labelled with anti-ZO1 antibody (red). Graph **(B′)** represents the quantification of the cell area of *n* > 350 cells from 3 independent experiments. Bars indicate the mean ± SEM. ****p < 0.0001, using unpaired *t*-test for a 2 by 2 comparison (Shear vs. Static) or One way Anova test (CTRL vs. KD cells). Scale bars: 20 µm. **(C)** Actin cytoskeleton in static condition (-Shear) and after shear stress (+Shear) was analysed by immunofluorescence in CTRL, KD_N1 and KD_N4 cells stained with phalloidin (grey). Graphs represent the quantification of the intensity of phalloidin per cell surface **(C′)** and that present at the membranes adjoined to tight junctions (co-labelling with ZO1) **(C”)**. Bars indicate the mean ± SEM of *n* > 350 cells from 3 independent experiments. ns: not significant, ****p < 0.0001, ***p = 0,0001, **p < 0.001, *p < 0,02, using unpaired *t*-test for a 2 by 2 comparison (Shear vs. Static) or One way Anova test (CTRL vs. KD cells). a.u.: arbitrary unit. Scale bars: 20 µm.

First, we analyzed ciliogenesis which was previously shown to be promoted by shear (Duan et al., 2008). As expected, the percentage of ciliated cells and cilium length increased in control cells cultured for 48 h upon shear compared to the static condition (Figures 3A−A″). In both KD_N1 and KD_N4 cell lines, impact of shear stress on ciliogenesis was also observed. Nevertheless, as seen in steady state (Figure 1D”), cilium length increase was more pronounced in KD_N1 cells under shear stress than in control or KD_N4 cells. These data suggest that loss of NPHP1 function alters shear stress-mediated regulation of cilium length.

As a second cellular process, we analyzed cell size shown to be reduced upon shear stress in other kidney cell types by the activation of autophagy and mTOR signaling pathways (Boehlke et al., 2010; Orhon et al., 2016; Miceli et al., 2020; Viau et al., 2020). We measured cell area using ZO1, a marker of cell junctions, and confirmed that in control mIMCD3 cells the cell size decreased upon shear stress by an average factor of 1.4. Similar effect was also seen in KD_N1 and KD_N4 cells (Figures 3B−B’) by an average factor of 1.3. These results indicated that mechanisms of cell size regulation upon prolonged shear stress were not affected by the absence of NPHP1 and NPHP4.

Finally, we explored the impact of shear stress on the remodeling of actin cytoskeleton. Shear stress has been shown to cause a redistribution of dense actin bands at the periphery of the cell, thus reinforcing the formation of both tight and adherens junctions, as well as to induce a disruption of basal stress fibers (Essig et al., 2001; Duan et al., 2008). We performed phalloidin staining to label actin fibers and noticed first, that, in static condition, like in steady state (Figure 1B), the actin network appeared altered, with an abundance of thick stress fibers in KD_N1 cells while stress fibers were sparse in KD_N4 cells. Quantification of the relative intensity of phalloidin, both in the cytoplasm and at the membrane, shows a 2-fold difference between control and KD_N4 cells, thus reinforcing this observation (Figures 3C–C″). Secondly, upon shear stress, a redistribution of actin was observed in control cells: actin was more homogeneously redistributed, with reinforcement at the membrane and in stress fibers (Figures 3C–C″). At the opposite, in KD_N1 cells, the stress fibers appeared thinner than in the static condition, suggesting a significant disruption of the fibers in these cells upon shear. In KD_N4 cells, an increase of the total amount of actin was observed, at both membrane and stress fibers. It is noteworthy that actin remodeling might be a process highly activated in KD_N4 cells, since these cells were initially poor of stress fibers.

Altogether, these results showed that overall, KD_N1 and KD_N4 cells do sense shear stress but exhibit inappropriate response to control cilium length and actin cytoskeleton remodelling, suggesting that NPHP1 and NPHP4 are required to ensure the proper dynamic response to shear.

### Transcriptomic analysis reveals cholesterol biosynthesis as a pathway modulated by shear stress

In order to explore the molecular changes occurring upon shear stress, we performed a transcriptomic study by RNA sequencing and compared static and shear conditions (48 h).

**FIGURE 4:**
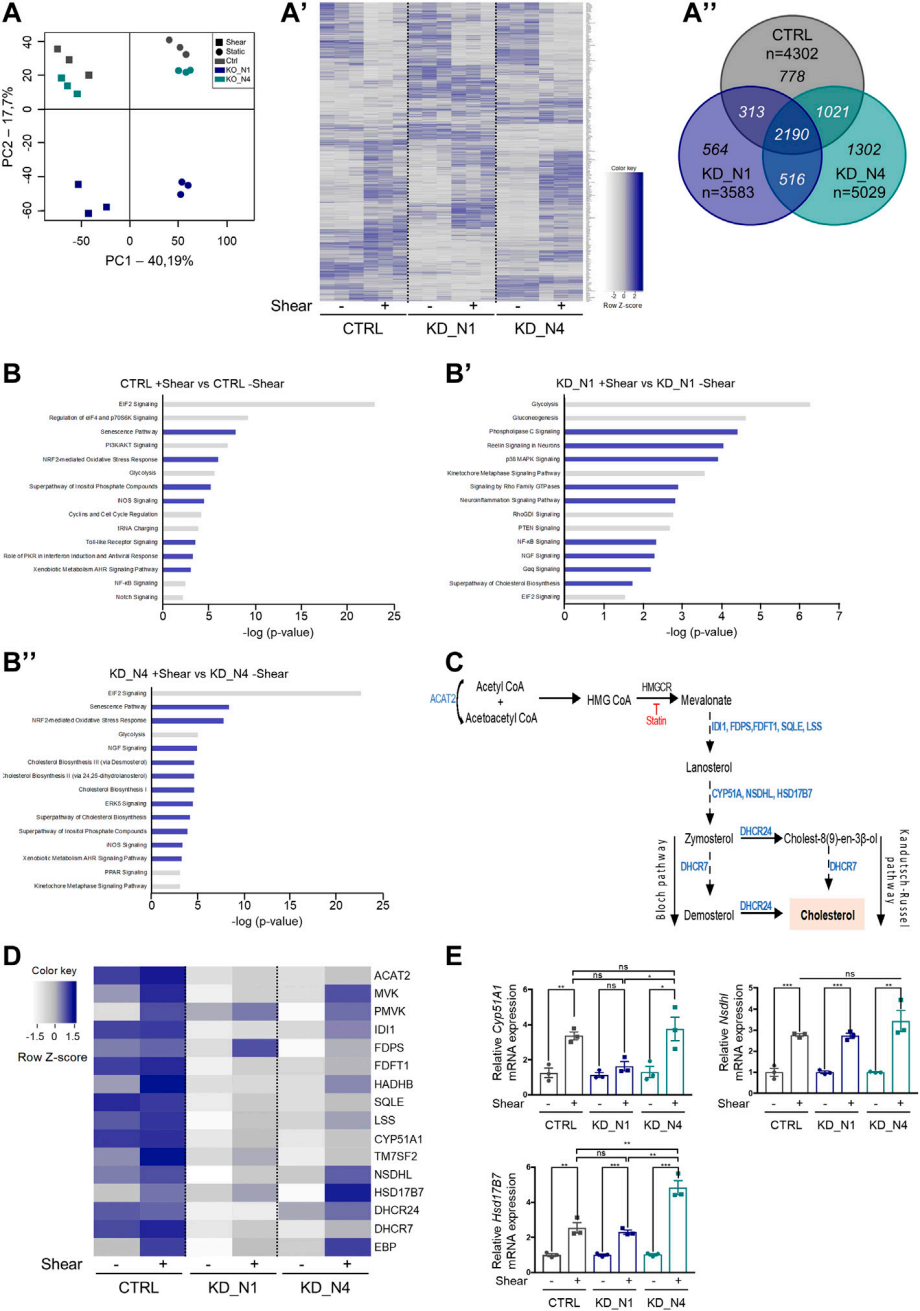
Transcriptomic analysis by RNA sequencing reveals that shear stress triggers cholesterol biosynthesis. **(A)** Principal component analysis (PCA) plot of the RNAseq data shows the samples in triplicates of each condition. Static conditions are represented by dots, shear conditions by squares. Heat map **(A′)** represents the gene expression of all genes modulated by shear stress as a function of cell type. Color map represents z-score with dark shades of blue indicating upregulated genes and light shades of blue indicating downregulated genes. Venn diagram **(A”)** represents the differentially expressed genes (fold change of 1.2) in CTRL, KD_N1 and KD_N4 cells between static and shear stress conditions. In italic are numbers of genes common to the three or two conditions, or unique to 1 cell type (*n* = 3 independent experiments). **(B)** Diagrams showing the 15 most deregulated signaling pathways following 48 h shear stress in CTRL **(B)**, KD_N1 **(B′)** and KD_N4 **(B”)** cells. Pathway enrichment analysis was performed using Ingenuity software. Pathways are ranked according to the *p*-value, with in blue, the upregulated signaling pathways (z-score >2) and in grey, the downregulated pathways (z-score < −1,5). **(C)** Simplified diagram of the key steps of the cholesterol biosynthesis pathway. It starts by the conversion of acetyl-CoA to HMG CoA by the HMG-CoA synthase, which is transformed into mevalonate by the action of HMG-CoA reductase (HMG-CR), a target of statin molecules. Mevalonate is then processed in several steps via the Bloch or Kandutsch-Russel pathways into cholesterol. In blue are indicated the enzymes involved in each step of the process. HMG CoA: 3-Hydroxy-3-Methylglutaryl-Coenzyme A. **(D)** Heat map representing the gene expression of enzymes of the cholesterol biosynthesis pathway that are identified by RNAseq to be modulated by shear. Color map represents z-score with dark shades of blue indicating upregulated genes and light shades of blue indicating downregulated genes. **(E)** RT-qPCR analyses of mRNA expression of *Cyp51A1*, *Nsdhl*, *Hsb17B7* normalized to the geometric mean expression of the house-keeping genes *Hprt*, *Sdha* and *Rpl13*. Bars indicate the mean ± SEM of 3 independent experiments. ns: not significant, ***p < 0,0008, **p < 0.007, *p < 0,03, using unpaired *t*-test for a 2 by 2 comparison (Shear vs. Static) or One way Anova test (CTRL vs. KD cells).

First, we performed an analysis of the principal components (PCA) to have an overview of our dataset and check its consistency. As shown in Figure 4A, we first observed that the triplicates of each condition are grouped together, confirming their homogeneity. Then, the principal component PC1 , differentiating shear (on the left) and static (on the right) conditions, is of 40%, while the second principal component, PC2 of 17%, explains the differences between KD_N1 on one side (bottom) and KD_N4 and control cells (CTRL) on the other side (top). This PCA shows that, overall, our samples are first distinguishable by the culture conditions, and second by the genotype. Of note, it reveals also that KD_N4 and control samples have a closer transcriptomic signature than with KD_N1 samples which is also visible on the heatmap (Figure 4A’).

In control cells, 48 h of shear stress induced a drastic change of gene expression compare to static condition, with the deregulation of a total of 4,302 genes, of which 2,236 were upregulated and 2,066 downregulated (fold change of 1.2) (Figure 4A”, Supplementary Table S1). Of note, *Nphp1* expression is slightly modulated by shear stress (fold change of 1.44), while *Nphp4* is not (fold change of 1.08).

To understand which signaling pathways were the most altered upon shear stress, we performed a pathway enrichment analysis using Ingenuity software (Qiagen) (Figure 4B, Supplementary Table S2). GO terms were selected according to the z-score and ranked according to the *p*-value. The z-score predicts the global status of activation or inhibition of a pathway by combining the expression patterns of the genes involved in the pathway. Upon 48 h of shear, the most regulated pathways in the control cell line were related to cell metabolism, with globally a decrease of mRNA translation (*EIF2 signaling, regulation of eiF4 and* p70 */S6K signaling, tRNA charging*), a promotion of cell cycle arrest (activation of *senescence pathway*, inhibition of *cyclins and cell cycle regulation*) and a moderation of energy production (decrease of *glycolysis*), with an inhibition of *PI3K/AKT signaling* that promotes metabolism, proliferation, growth, angiogenesis and cell survival. As previously reported, shear stress induces the production of reactive oxygen species (ROS) (Zheleznova, Yang, and Cowley, 2016a) and nitric oxide (NO) (Cabral, Hong, and Garvin, 2010) (activation of *iNOS pathway*), which helps to cope the stress and modulates glucose and lipid metabolism. Together with that, *NRF2-mediated oxydative stress response* pathway that protects the cell against ROS and/or NO was activated. Several GO terms suggested an activation of inflammation (*Toll-like receptor signaling, NF-kB signaling*)*,* which has been previously demonstrated as a consequence of shear stress (Miravète et al., 2011, Miravète et al., 2012). Finally, amongst the top modulated pathways, there was also the phosphoinositide-related signaling which induces cytoskeletal changes and actin remodeling, and plays a role in clathrin-mediated endocytosis, vesicle trafficking, membrane dynamics, autophagy, cell division/cytokinesis and cell migration.

In KD_N1 cells, 3,583 genes were deregulated by shear stress, of which 1,968 were upregulated and 1,615 were downregulated. In KD_N4 cells, 5,029 genes were deregulated by shear stress, of which 2,754 were upregulated and 2,275 were downregulated (fold change of 1.2) (Figure 4A’, Supplementary Table S1). Globally, as the PCA and the heatmap underlined it, there were more genes commonly shared by KD_N4 and CTRL than between KD_N1 and each of the two other conditions, suggesting once again that KD_N4 and CTRL might have a close transcriptomic signature in response to shear (the most common pathway being the *EIF2 signaling* that is highly significant and has a high z-score in both KD_N4 and CTRL conditions). Analyzing the GO terms, we observed that some of the signaling pathways highlighted in the control condition were also modulated in both KD_N1 and KD_N4 cell lines, notably the decrease of energy production (*glycolysis*) and of mRNA translation (*EIF2 signaling*), and the activation of inflammation (*neuroinflammation pathway*) (Figures 4B’, B″). The production of NO and/or the activation of pathways protecting against ROS/NO were also amongst the top pathways modulated by shear in KD_N1 and KD_N4 cells.

In contrast, we observed pathways more specific to KD_N1 and KD_N4 conditions, notably the upregulation of the *signaling of Rho family GTPases* (KD_N1: z-score of 4.06, -log (pval) of 2.88; KD_N4: z-score of 2.83, -log (pval) of 4.96) along with the downregulation of the counteracting *RhoGDI signaling* (KD_N1: z-score of −2.6, -log (pval) of 2.76; KD_N4: z-score of −1.81, -log (pval) of 2.11) (Figures 4B’, B″, Supplementary Table S2). Rho GTPases are central regulators of actin reorganization and consequently function in cellular processes such as cell migration, wound healing, cell adhesion, cell polarity, membrane trafficking and cytokinesis (Van Aelst and Symons, 2002). This result is in line with the phenotype observed in KD_N1 and KD_N4 cells, with a perturbed actin cytoskeleton at steady state and its important remodeling upon shear stress. Another interesting pathway present in KD_N4 line is the *PPAR signaling* (KD_N4: z-score of −1.81, -log (pval) of 2.95), that was mostly downregulated. PPAR stands for peroxisome proliferator-activated receptors, which are nuclear receptors activated by fatty acid derivatives (Varga, Czimmerer, and Nagy, 2011). It regulates energy homeostasis: when activated, it leads to the stimulation of the fatty acid oxydation to produce energy. PPAR signaling has also a role in cholesterol metabolism, and tends to enhance the cholesterol efflux to lower its concentration.

Finally, in both KD cell lines, the *cholesterol biosynthesis pathway* appeared in the top 15 pathways modulated by shear stress, and we chose to study this further. Indeed, contrary to previous reports that showed that membrane cholesterol modulates shear stress-induced signaling pathways, thus participating in the processes of mechanosensation (Ferraro et al., 2004; Zhang et al., 2011; Yamamoto and Ando, 2015), our data indicate here that cholesterol biosynthesis could itself be regulated by shear stress.

Cholesterol is synthesized in the endoplasmic reticulum by the concerted action of more than 30 enzymes organized in the mevalonate pathway, that transforms acetyl-CoA in lanosterol, which is then converted in several final steps into cholesterol (Figure 4C; Ačimovič and Rozman, 2013). The limiting reaction in cholesterol biosynthesis is the conversion of HMG CoA to mevalonate, catalyzed by HMG-Co reductase (HMG-CR). This enzyme is tightly regulated at transcriptional and post-transcriptional levels and is the pharmacological target of statins, the most widely used family of cholesterol-lowering drugs. In a heatmap, we plotted the genes involved in this pathway that were highlighted by the RNAseq data (Figure 4D). The genes are classified according to the position of the enzymes they encode in the cholesterol biosynthesis pathway. We can notice that the majority of the genes code for enzymes involved in the last steps of the biosynthesis pathway, notably the steps leading to the synthesis of cholesterol from lanosterol (Figure 4D). The heatmap shows that in control cells, the biosynthesis was active in static condition and slightly stimulated upon shear. In both KD_N1 and KD_N4 cells, expression of cholesterol genes was initially very low, and then activated upon shear, which was more obvious for KD_N4 cells. To validate the transcriptomic data, we selected three of the modulated genes (*Cyp51A1, Nsdhl, Hsd17B7*) and quantified their expression by RT-qPCR in static and shear stress conditions. Overall, shear stress induced an increase of expression of all three tested genes, by a factor of 2 to 4 (Figure 4E), thus confirming the RNAseq data.

Altogether, RNAseq analysis, further confirmed by RT-qPCR analysis, allowed us to identify a novel pathway, the cholesterol biosynthesis, as a pathway modulated by shear stress in control, KD_N1 and KD_N4 cell lines.

### Fluid shear stress triggers cholesterol biosynthesis independently of NPHP1 and NPHP4

**FIGURE 5:**
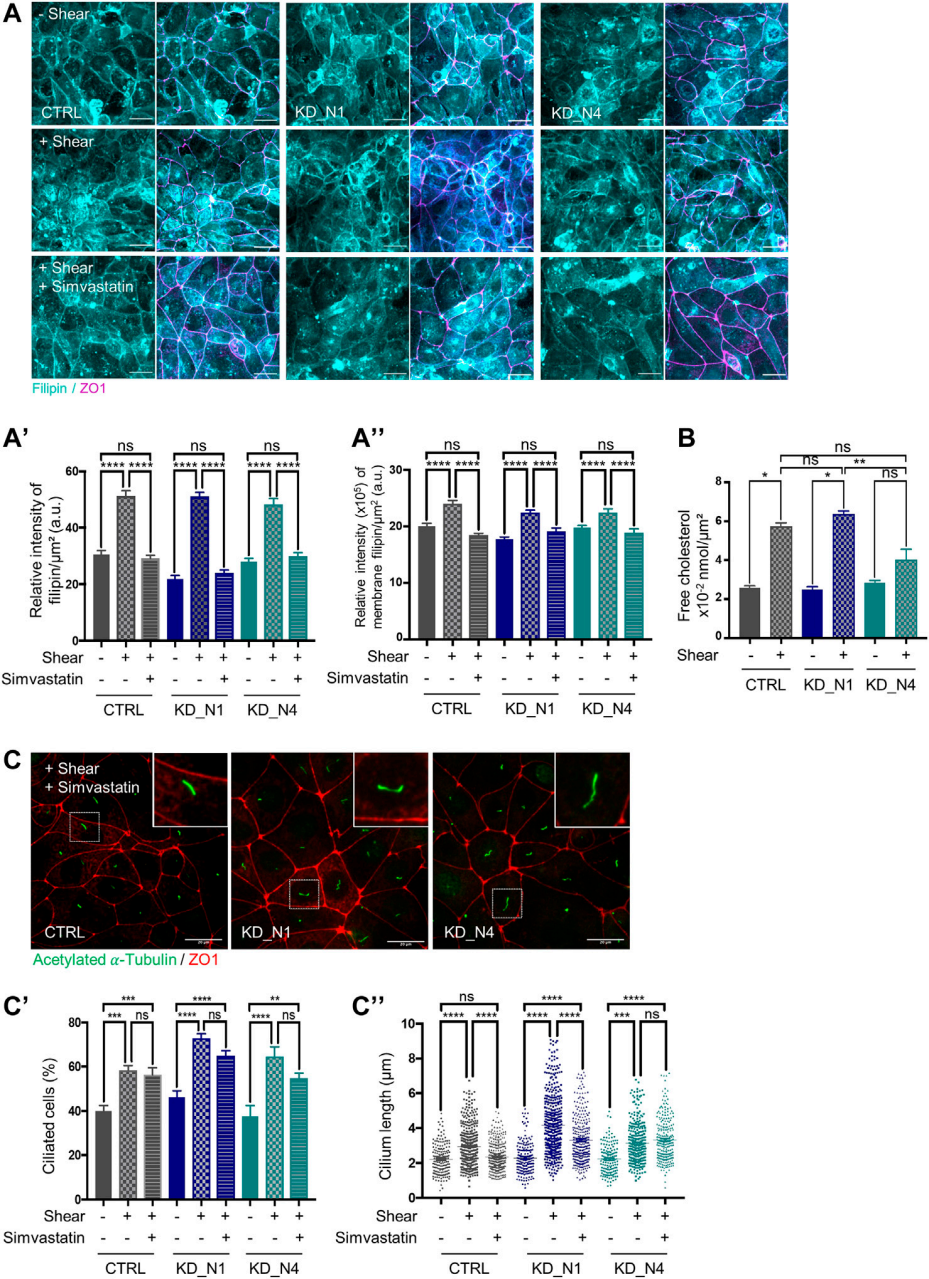
Fluid shear stress triggers cholesterol biosynthesis independently of NPHP1 and NPHP4. **(A)** Cellular cholesterol content in static condition (–Shear) and after shear stress, without (+Shear) or with 5 µM simvastatin (+Shear + Simvastatin), was analysed by immunofluorescence in CTRL, KD_N1 and KD_N4 cells stained with the fluorescent probe filipin (cyan). Graphs represent the quantification of the filipin intensity per cell surface **(A′)** or that present at the membranes adjoined to tight junctions (co-labelling with ZO1) **(A”)**. Bars indicate the mean ± SEM of *n* > 350 cells from 3 independent experiments. ns: no significant, ****p < 0.0001, ***p < 0.0004, *p < 0.02, using One Way Anova test. a.u.: arbitrary unit. Scale bars: 20 µm. **(B)** Free cholesterol extraction and analysis by Gas Chromatography–Mass Spectrometry (GC-MS). Cells from microslides were resuspended with methanol and lipids were extracted and injected into the GC-MS. The graph represents the total extract cholesterol in nmol per cell surface. Bars indicate the mean ± SEM of 4 independent experiments. ns: not significant, **p < 0.007, *p < 0,02 using Mann Whitney for a 2 by 2 comparison (Shear vs. Static) or Kruskal-Wallis test (CTRL vs. KD cells). **(C)** Ciliogenesis after shear stress with 5 µM simvastatin was analysed by immunofluorescence in CTRL, KD_N1 and KD_N4 cells labelled with anti-acetylated α -tubulin antibody (cilia, green) and anti-ZO1 antibody (red). Higher magnification images of the outlined area are shown on the right. Graphs represent the quantification of the percentage of ciliated cells **(C′)** and cilium length **(C′′)** of n > 350 cells from 3 independent experiments. Bars indicate the mean ± SEM. ns: not significant, ****p < 0.0001, ***p < 0,0002, **p < 0.002, using One Way Anova test. Scale bars: 20 µm.

To assess the effect of shear stress on the amount of intracellular cholesterol, we performed a staining with filipin, a fluorescent compound from *Streptomyces filipinensis* that specifically binds to free cholesterol (Wüstner, 2012). In control cells, as well as in KD_N1 and KD_N4 cells, there was an increase in the amount of cholesterol content upon shear stress (Figures 5A, A’). A similar increase of cholesterol amount at the apical cell membranes was also observed in all cell lines (Figure 5A”) when using co-labelling with ZO1. This observation was confirmed by extraction and analysis of free cholesterol by Gas Chromatography-Mass Spectrometry (GC-MS) (Figure 5B).

To evaluate the contribution of the cholesterol biosynthesis pathway in this increase, cells were treated with simvastatin, an inhibitor of the HMG-Co reductase (HMG-CR), for 48 h during shear stress. In control and KD cells, a decrease of the amount of cholesterol was observed after treatment (Figures 5A–A”). Thus, we concluded that shear stress does activate cholesterol biosynthesis that is correlated with an increase of cellular cholesterol amount, and this process is independent of NPHP1 and NPHP4.

Then, a step further, we evaluated whether the increase in cholesterol participates to the cellular responses to shear stress, and we analysed ciliogenesis. Blockade of the cholesterol biosynthesis pathway by statins has already been shown to decrease the percentage of ciliated cells and cilium length (Maerz et al., 2019). Here, we found that simvastatin treatment during shear stress did not affect the percentage of ciliated cells (Figures 5C, C′); however, it decreased cilium length to the level of static condition in control cells whereas it had a slight to no effect in KD_N1 and KD_N4 cells (Figures 5C–C″). Altogether these data suggest that the shear stress-induced regulation of cilium length is dependent on cholesterol synthesis and that this cholesterol-dependent response is altered in KD_N1 and KD_N4 cells.

### Fluid shear stress triggers cholesterol biosynthesis and uptake via SREBP2

**FIGURE 6:**
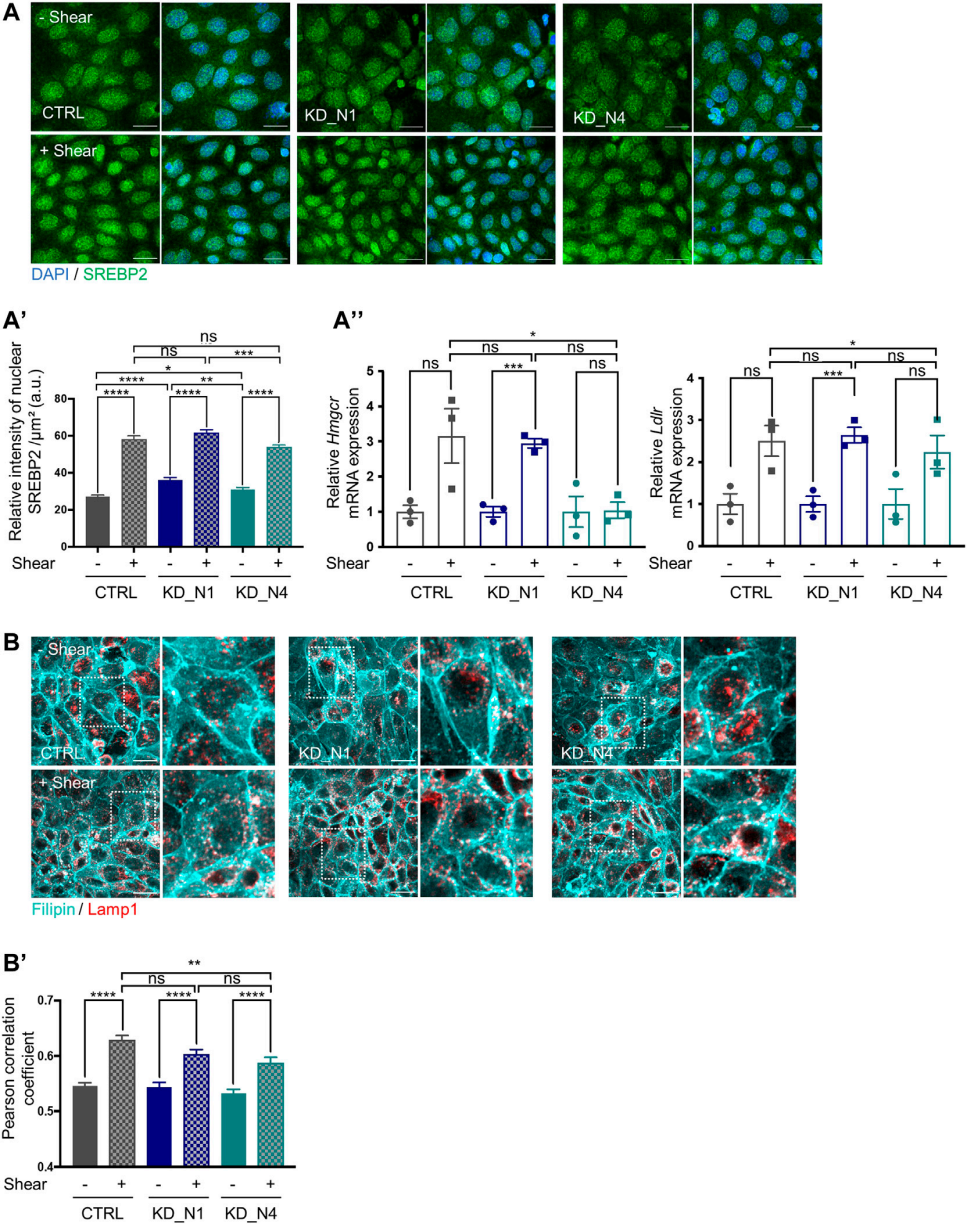
Fluid shear stress triggers cholesterol biosynthesis and uptake via SREBP2. **(A)** SREBP2 localization in nucleus, in static condition (–Shear) and after 48 h shear stress (+Shear) was analyzed by immunofluorescence in CTRL, KD_N1 and KD_N4 cells labelled with anti-SREBP2 antibody (green) and DAPI (nuclei, blue). Graph represents the quantification of the nuclear SREBP2 intensity per cell surface **(A′)**. Bars indicate the mean ± SEM of *n* > 350 cells from 3 independent experiments. ns: no significant, ****p < 0.0001, ***p < 0,0003, **p < 0.003, *p < 0,03, using unpaired *t*-test for a 2 by 2 comparison (Shear vs. Static) or One way Anova test (CTRL vs. KD cells). RT-qPCR analysis **(A′′)** of mRNA expression of *Hmgcr* and *Ldlr*, normalized to the geometric mean expression of the house-keeping genes *Hprt*, *Sdha* and *Rpl13*. Bars indicate the mean ± SEM of 3 independent experiments. ns: not significant, ***p = 0,0006, **p = 0,0033, *p < 0,04, using unpaired *t*-test for a 2 by 2 comparison (Shear vs. Static) or One way Anova test (CTRL vs. KD cells). **(B)** Cholesterol localisation in lysosomes, in static condition (–Shear) and after shear stress (+Shear) was analysed by immunofluorescence in CTRL, KD_N1 and KD_N4 cells labelled with the fluorescent probe filipin (cyan) and with anti-LAMP1 antibody (red). Higher magnification images of the outlined area are shown on the right. Graph represents the Pearson’s correlation coefficient **(B′)** that evaluates the colocalization of filipin and LAMP1 staining. Bars indicate the mean ± SEM of *n* = 10 fields from 3 independent experiments. ns: no significant, ****p < 0.0001, **p < 0.004, using unpaired *t*-test for a 2 by 2 comparison (Shear vs. Static) or One way Anova test (CTRL vs. KD cells).

Cellular cholesterol is maintained at homeostatic levels by the concerted action of transcriptional and post-transcriptional mechanisms. At transcriptional level, the SREBP/SCAP/INSIG complex modulates cholesterol synthesis and uptake. When intracellular cholesterol levels are low, the mature form of SREBP translocates to the nucleus where it binds to the transcriptional regulator SRE (Serum Response Element) sequences in the regulatory region of the target genes, *Hmgcr* and *Ldlr* (Low Density Lipoprotein Receptor), thereby activating their transcription (Ikonen, 2008). It thus results into the activation of cholesterol synthesis through increased levels of HMG-CR and the promotion of LDL uptake by LDL receptors. To study the regulation of intracellular cholesterol levels via SREBP, we analysed its subcellular localisation in nuclei, under static and shear stress conditions, by immunofluorescence. We observed an increase in the amount of nuclear SREBP in all cell lines after shear stress (Figures 6A, A'), as well as an increase of mRNA expression of the *Hmgcr* and *Ldlr* genes in control and KD_N1 cells, by RT-qPCR (Figure 6A”). In KD_N4 cells, shear-stress mediated increase of *Hmgcr* gene expression could not be observed (Figure 6A”); however, the increased expression of downstream enzymes (Figures 4D,E), including MVK that is also regulated by SREBP2 (Vergnes et al., 2016), might compensate the lack of *Hmgcr* gene expression regulation. These results indicated that shear stress-mediated activation of cholesterol biosynthesis pathway is ensured by SREBP/SCAP/INSIG complex; they also indicated that cholesterol could also be imported from the extracellular compartment through LDL uptake. To confirm this, we studied the lysosome compartment using co-staining with LAMP1. Upon 48 h shear stress, we observed that, whatever the cell line, shear stress induced an accumulation of cholesterol in lysosomes, as illustrated by the increase of Pearson’s correlation between filipin and LAMP1 staining. This result suggested that the transport of cholesterol from lysosomes was more intense upon shear stress, and this was independent of NPHP1 and NPHP4 (Figures 6B, B′).

Altogether, these results clearly showed that shear stress induced cholesterol biosynthesis and uptake in mIMCD3 cells, through the transcriptional regulation of SREBP protein.

## Discussion

In the present study, we focused on the putative roles of nephrocystins NPHP1 and NPHP4, two proteins involved in nephronophthisis, in the processes of flow sensation. Altogether, our data show that the loss of function of Nphp1 and Nphp4 leads to altered response to shear stress, with an inappropriate immediate calcium response, a defective regulation of cilium length (for Nphp1) and an impaired actin network remodeling. Since *Nphp1* and *Nphp4* expression is barely or not activated upon shear stress, we presume that Nphp1 and Nphp4 have indirect roles in flow sensation that are discussed below. In addition, our work allowed us to highlight for the first time the cholesterol biosynthesis and uptake pathways as being regulated by shear stress in kidney cells. We showed that upon shear stress, both cholesterol content and cellular distribution are modulated, processes in which NPHP1 and NPHP4 proteins do not play a central role. It is noteworthy that the lack of total loss of expression of Nphp1 and Nphp4 proteins in our cellular models might have impede the observation of stronger involvement of both proteins in flow sensation response and cholesterol biosynthesis regulation.

As a first read-out, we analyzed the immediate calcium response. It is triggered by various mechanisms, and notably an initial calcium entry through mechanosensitive channels or GPCR located at the plasma membrane and at ciliary membrane. This event activates the phospholipase C protein that cleaves the phosphoinositide PIP2 into DAG and IP3, which in turn activates the release of calcium from the intracellular store, the endoplasmic reticulum (ER). By using a calcium biosensor, we showed that the increase of intracellular calcium level upon shear stress sensation happens faster in KD cells than in control. This result indicates that KD cells are more responsive to shear stress, which can be due to an over-activation of mechanosensitive channels or GPCR, or to an inappropriate control of calcium release from ER. The impact of this exacerbated calcium response in KD_N1 and KD_N4 cells under shear stress remains to be evaluated, but it may presumably alter cellular calcium homeostasis with detrimental consequences for cell function as differentiation, proliferation or apoptosis (Berridge, Lipp, and Bootman, 2000).

Interestingly, it has been shown that cilium length is a factor of shear stress sensitivity. The longer the cilia are, the more prone they are to bend, thus contributing to the activation of mechanosensitive channels present at its surface (Abdul-Majeed, Moloney, and Nauli, 2012; Chikamori et al., 2020). Hence, since we showed that KD cells have elongated cilia at steady state, we can hypothesize that cilia contribute to the sensitivity of KD cells to shear stress. Of note, elongated cilia were also detected *in vivo* in the renal tubules of both *Nphp1* ^−/−^ mouse model and biopsies from patients with *NPHP1* mutations (Garcia et al., 2022). Hence, excessively long cilia, further exacerbated by shear stress, might fail to act as efficient mechanosensor, resulting as short cilia do, in improper regulation of cellular processes and in kidney cyst development (Dell, 2015).

We also showed that KD cells, and especially KD_N1, are unable to correctly regulate cilium length upon shear stress. One of our recent studies showed that alteration of cilium length in *NPHP1* models was related to decreased ciliary localisation of adenylate cyclases and cAMP production, rescued by the treatment with prostaglandins (Garcia et al., 2022), which release is stimulated by shear stress. Hence, we can hypothesize that alteration of immediate response to shear stress in KD cells can contribute to an improper modulation of prostaglandin signaling. Another parameter that controls cilium length is the actin cytoskeleton (Smith, Lake, and Johnson, 2020). We showed that the actin network is impaired in KD cells lines, which was further underlined by our transcriptomic analysis that revealed an upregulation of the RhoGTPase pathway along with the downregulation of the RhoGDI (Rho GDP-dissociation inhibitor) signaling. Among the members of the Rho GTPase family is Cdc42, which gene is upregulated in KD cells. It has been shown that the Cdc42 effectors, p130Cas, Par6 and ACK1, as well as the associated actin network proteins, filamin and tensin, interact with NPHP1 and NPHP4 (Donaldson et al., 2000; Benzing et al., 2001; Mollet et al., 2005; Eley et al., 2008; Delous et al., 2009). This suggests that NPHP1 and NPHP4 can modulate the activation of Rho GTPases and thus participate to the reorganization of the actin cytoskeleton, as well as the related cellular processes, including ciliogenesis.

To our knowledge, the transcriptomic analysis we performed at 48 h shear stress in the first of its kind. Previous RNAseq studies on kidney cells (mIMCD3 or PTEC) subjected to short shear stress exposure (3 h or 6 h) highlighted pathways related to cell-cell and cell-matrix interactions, as well as cytoskeleton remodeling. More specifically, the MAPK, TNF and TGF-b signaling pathways were upregulated while JAK-STAT or Rho were downregulated (Mohammed et al., 2017; Kunnen et al., 2018a). In our study, after 48 h of shear stress, these pathways were not differentially modulated between static and shear conditions; instead we brought out pathways that regulate glucose and lipid metabolism, induce inflammation or protect against reactive oxygen species and nitric oxide, released upon shear stress.

The most remarkable pathway that is modulated by shear stress at 48 h is the cholesterol biosynthesis pathway. Very few studies report a link between cholesterol and shear stress in the kidney. Cellular cholesterol composition has been shown to affect COX2 expression and shear stress-mediated PGE2 release via a p38-dependent mechanism (Liu et al., 2015). Cholesterol can also repress the expression of genes involved in sodium and water regulation, leading to increased Na^+^ and water retention (Repetti et al., 2019). We show here for the first time the direct link between cholesterol biosynthesis and shear stress, in which NPHP1 and NPHP4 do not seem to be involved. We showed that shear stress induces an increase of cholesterol biosynthesis after 48h, via the activation of the SREBP/SCAP/INSIG complex, which results into a stronger proportion of cholesterol at the membranes adjoined to junctions as well as in lysosomes. Modulation of cholesterol content upon shear stress seems important for the cell responses. Indeed, we showed that treatment with simvastatin suppressed shear stress-induced cilium length regulation in control cells and partially in KD_N1 and KD_N4 cells. Such observation has been reported in both cultured cells and zebrafish, in which absence of cholesterol resulted in cilium frequency and length decrease (Maerz et al., 2019). Hence, treating KD cells with simvastatin could dampen their excessive cilium length and their sensitivity to shear stress.

To conclude, although our study has not identified a direct role of NPHP1 and NPHP4 in regulation of cholesterol content in mIMCD3 cells exposed to shear stress, we showed that targeting the cholesterol pathway in KD_N1 and KD_N4 cells influences cilium length regulation, potentially offering a new therapeutical approach for NPH patients.

## Materials and methods

### CRISPR/Cas9-mediated invalidation of *Nphp* genes in mIMCD3 cells

To avoid off-target effects, the strategy of double Cas9 nickase was used. For that, two guide RNAs were designed using CRISPOR online tool (http://crispor.tefor.net; Concordet and Haeussler, 2018), to target an out-of-frame exon of each gene, i.e., exon 10 (#10) for *Nphp1* (NM_001128178) and exon 4 (#4) for *Nphp4* (NM_015102). Guides were cloned into pSpCas9n (BB)-2A-GFP/mCherry plasmids expressing nickase Cas9 along with GFP (gift from Feng Zhang (Ran et al., 2013), Addgene plasmid #48140) or mCherry following the protocol described in (Reilly et al., 2019).

After cotransfection of both GFP and mCherry plasmids, double positive mIMCD3 cells were sorted by flow cytometry prior to clonal dilution. Genomic DNA of each single clones was extracted using QuickExtractTM DNA Extraction Solution (Tebu-bio, QE09050) and the genotype was determined by Sanger sequencing.

### Cell culture and flow chamber

mIMCD3 cells were cultured in DMEM/F-12 (Gibco^®^) supplemented with 10% FBS, glutamine and penicillin/streptomycin. For culture in flow condition, cells were seeded at confluence (200,000 cells/cm^2^) in closed perfusion chambers (Microslide I 0.6 Luer, Ibidi) and cultured for 1 day at 37°C and 5% CO_2_. Cells were then either incubated in static conditions or exposed to a shear stress of 1 dyn/cm^2^ for 48 h. Medium was refreshed twice a day for static conditions. For shear stress experiments, the chamber was connected to a computer-controlled set-up containing an air-pressure pump and a two-way switching valve (Ibidi pump system), and 10 mL of cell culture medium was pumped unidirectionally between two reservoirs through the flow channel at a rate corresponding to a shear stress of 1 dyn/cm^2^. For statin treatment, 5 μM of Simvastatin was added to the 10 mL of cell culture medium in both reservoirs connected to the flow chambers during the shear stress experiment.

### Immunofluorescence

Cells were cultured on coverslips or in microslides, washed in PBS and fixed in 4% PFA at room temperature for 20 min. After a wash in PBS and 1 h blocking in PBS; BSA 1%; Tween 0.1%, cells were incubated with the appropriate primary antibodies overnight at 4°C: acetylated α -Tubulin (1:1,000; Sigma, T6793), γ -Tubulin (1:200; Sigma, T6557), NPHP1 (1:100; Bicell scientific, 90001), ZO1 (1:100; Invitrogen, 33–9,100), Lamp1 (1:100; Abcam, 24170), SREBP-2 (1:200; Santa Cruz, sc-13552) followed by secondary antibodies (Alexa Fluor^®^ 488, 555 or 647, 1:200; Thermo Fisher Scientific) for 30 min at room temperature. The intercalated dyes used were Alexa Fluor™ 647 Phalloidin (1:300; Thermo Fisher Scientific, A22287) and DAPI (1:2000; Thermo Fisher Scientific, 62247). All antibodies were diluted in blocking buffer. For filipin staining, cells were washed in PBS three times and incubated in the filipin solution at a concentration of 50 μg/mL (Sigma Aldrich, F-9765) for 30 min at room temperature. Coverslips and microslides were mounted in Mowiol media. Image acquisition was performed with a Yokogama CSU-X1 spinning disk microscope (63×, NA 1.4) (filipin staining) or a Leica SP8 laser scanning confocal microscope (63×, NA 1.4). Fiji software (http://fiji.sc/) was used for all quantifications, with the JACoP plugin for Pearson correlation coefficient analysis. Measurements of cilium length was done with Imaris software (Oxford Instruments).

### Calcium measurements

Cells were seeded at a density of 200,000 cells/microslide with a growth area of 2.5 cm^2^ and grown for 1 day prior to transfection. Cells were then transfected with 500 ng of R-GECO calcium biosensor plasmid (Addgene plasmid # 45494) using Lipofectamine 2000 (Thermo Fisher Scientific, 11668-019) for 48 h and cultured with phenol red-free medium. Images were acquired on a Nikon Ti-E inverted microscope equipped with a temperature chamber set at 37°C, at the ×40 objective (NA 1.4). The shear stress of 1 dyn/cm^2^ was applied after 1 min of acquisition over a total period of 5 min. R-GECO signal intensity was normalized to background $\left(\Delta F/F0\right)$ *,* where F0 is the average intensity over the first 30 s, were measured for each cell over time using ImageJ software. The response time following shear stress initiation $\left(\Delta t\right)$ was evaluated for all analyzed cells.

### RNAseq analysis

Total RNA from 3 different experiments were isolated using the RNeasy Kit (QIAGEN) including a DNAse treatment step. RNA quality was assessed by capillary electrophoresis using High Sensitivity RNA reagents with the Fragment Analyzer (Agilent Technologies) and the RNA concentrations were measured by both spectrophometry using the Xpose (Trinean) and Fragment Analyzer capillary electrophoresis. RNAseq libraries were prepared starting from 100 ng of total RNA using the universal Plus mRNA-Seq kit (Nugen) as recommended by the manufacturer. The oriented cDNA produced from the poly-A+ fraction were sequenced on a NovaSeq6000 from Illumina (Paired-End reads 100 bases +100 bases). A total of ∼50 millions of passing filter paired-end reads was produced per library. FASTQ files were mapped to the ENSEMBL reference (Mouse GRCm38/mm10) using Hisat2 and counted by the Counts function of the R Subread package (http://www.r-project.org/). Normalizations of read counts and group comparisons were performed by the statistical methods Deseq2, Voom and edgeR. Flags were calculated from counts normalised to the mean coverage. All normalised counts <20 were considered background (flag 0) and ≥20 as signal (flag = 1). P50 lists used for the statistical analysis regroup the genes showing flag = 1 for at least half of the compared samples. The results were filtered at a *p*-value ≤0.05 and a fold-change of 1.2. Gene lists common to all three methods were uploaded to Ingenuity Pathway Analysis (Qiagen) to determine canonical pathways differentially regulated under shear stress. The PCA has been performed using “prcomp” function in R and the 2 first dimensions were plotted. The clustering has been performed using “dist” and “hclust” functions in R, using Euclidean distance and Ward agglomeration method. Bootstraps have been realized using “pvclust” package in R, with the same distance and agglomeration method, using 1,000 bootstraps. Results for cholesterol biosynthesis genes are shown as a heat map made with the R package ctc: Cluster and Tree Conversion and imaged by Java Treeview software (Java Treeview—extensible visualization of microarray data).

### RT-qPCR analysis

Total RNA was reverse transcribed using SuperScript II Reverse Transcriptase (LifeTechnologies) according to the manufacturer’s protocol. Quantitative PCR was performed with iTaqTM universal SYBR^®^ Green Supermix (Bio-Rad) on a CFX384 C1000 Touch (Bio-Rad). *Hprt*, *Rpl13* and *Sdha* were used as normalization controls (Vandesompele et al., 2002). Each biological replicate was measured in technical duplicates. The primers used for RT-qPCR are listed in Supplementary Table S3.

### Free and esterified cholesterol analysis by GC/MS and LC-MS/MS

Deuterated internal standards were purchased from Avanti Polar Lipids (Alabaster, AL, United States). LC/MS grade or UPLC grade solvents were used without further purification and obtained from Merck KGaA (Darmstadt, Germany).

Cells on microslides were resuspended with 3 times 150ul methanol. Free and esterified sterols were extracted using a modified Bligh and Dyer method. Resuspended cells (total volume of 450ul) were supplemented with deuterated internal standards (cholesterol d7 and CE (18:1-d7)) and lipids extracted with 1.5 mL chloroform and 300ul methanol (total methanol fraction 750ul). Phase separation was triggered by addition of 450 µL of ammonium carbonate (250 mM). Extracted lipids were dried and resuspended in LC/MS solvent.

Cholesterol esters were quantified by LC-ESI/MS/MS using a Prominence UFLC (Shimadzu, Tokyo, Japan) and QTrap 4,000 mass spectrometer (AB Sciex, Framingham, MA, United States). Sample was injected into an Ascentis Express C18 column (150 × 2.1 mm, 2.7 µm) (Merck Group). Mobile phases consisted of A) acetonitrile/water (60:40 v/v) and B) isopropanol/acetonitrile (90:10 v/v) supplemented with 10 mmol/L ammonium formate and 0.1% formic acid. Lipid species were detected using scheduled multiple reaction monitoring.

For the quantification of free cholesterol, previously extracted lipids were dried and derivatized with BSTFA/TMCS (10%) for 1 h at 80°C. Silylated cholesterol was quantified by GC/MS (Trace ISQ, ThermoFisherScientific) using split mode injection at 250°C and separation on a 50 m × 0.25 mm, 0.25 µm DB-5MS capillary column. Cholesterol was detected by electronic ionization in SIM mode.

### Statistical analysis

Data from 3 independent experiments were expressed as means ± SEM. After performing a Shapiro–Wilk normality test, differences between groups were evaluated using unpaired *t*-test or Mann Whitney test when only two groups were compared or when testing more comparisons by One Way Anova test or Kruskal–Wallis test. The statistical analysis was performed using GraphPad Prism V7 software.

## Data Availability

The original contributions presented in the study are publicly available. This data can be found here: https://www.ebi.ac.uk/biostudies/studies/S-BSST1213 using the accession number S-BSST1213.
