# Supplementary figures and images for: Fluid shear stress triggers cholesterol biosynthesis and uptake in inner medullary collecting duct cells, independently of nephrocystin-1 and nephrocystin-4
Source: Front Mol Biosci. 2023 Oct 17;10:1254691. doi: 10.3389/fmolb.2023.1254691 (PMC10616263; doi:10.3389/fmolb.2023.1254691)

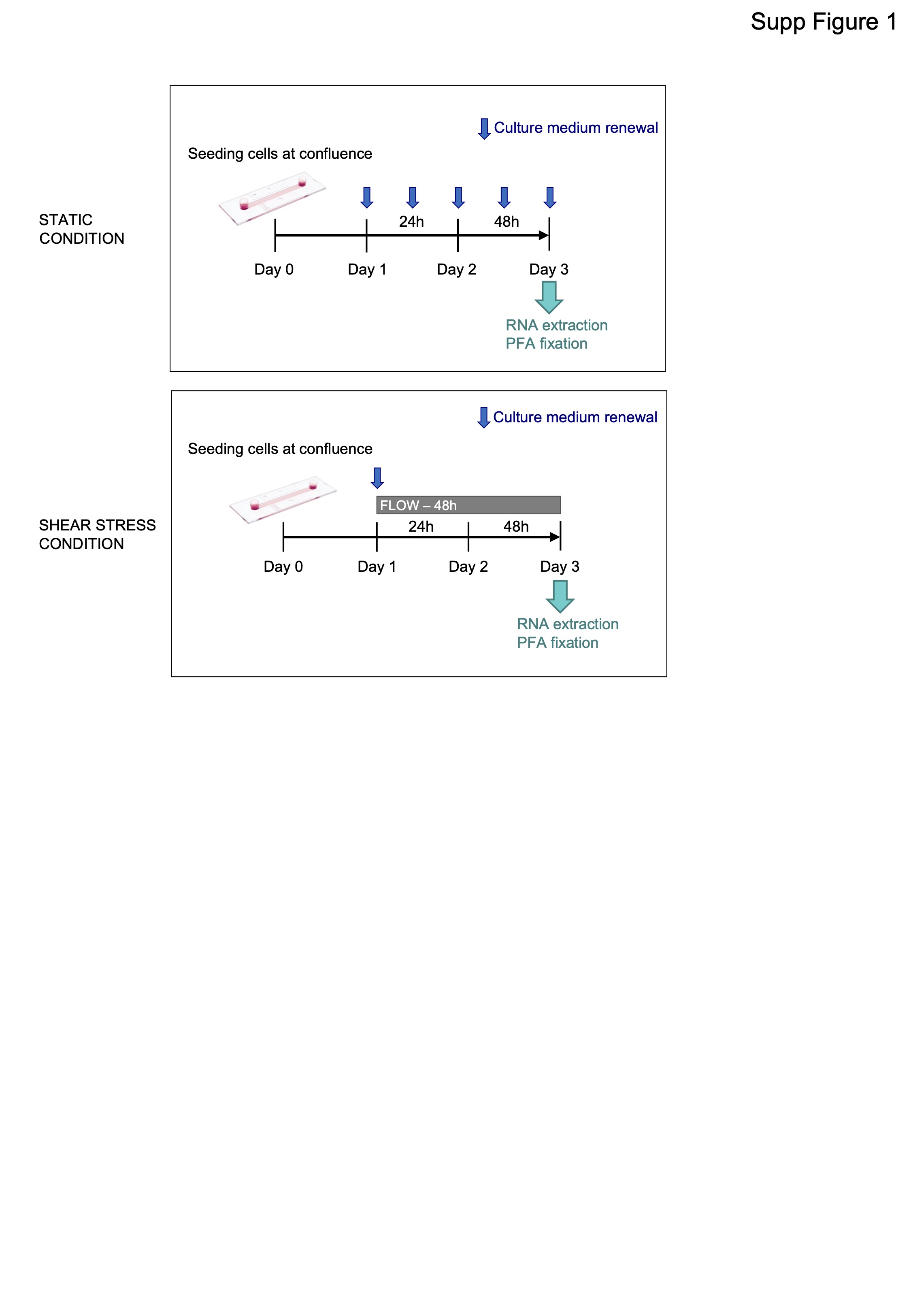

Supplement: Supplementary file 6 [file Image1.jpg]
